# Disability & Diversity studies as a professional basis for diversity-aware education and training in medicine

**DOI:** 10.3205/zma001316

**Published:** 2020-03-16

**Authors:** Susanne Dungs, Christine Pichler, Ralf Reiche

**Affiliations:** 1 FH Kärnten, Disability & Diversity Studies für Berufstätige, Klagenfurt, Austria; 2 FH Kärnten, Gesundheitsmanagement, Feldkirchen in Kärnten, Austria

**Keywords:** disability & diversity studies, disability, diversity, intersectionality, diversity in medical practice

## Abstract

The “Disability and Diversity Studies“ (DDS) are research fields which, similar to social work, deal with social inclusion and exclusion processes. Dimensions of disability and diversity can lead to disadvantages and inequalities in the individual life and social coexistence of people. The DDS examine these inequalities and identify intersectional relationships between diversity categories. The concept of intersectionality opens up the view of the restriction of diversities, which can lead to the intensification of inequalities and multiple discriminations: e. g., in the case of being a woman and member of an ethnic minority. The starting point is therefore not the difference category per se, but the intersection of several categories [5]. This knowledge of categorizing classification and exclusion in their intersectionality is fundamental for the dissolution of social, societal, political and economic inequality. The DDS bachelor's program at the Carinthia University of Applied Sciences, which combines Disability and Diversity Studies, focuses on these research areas and develops practical solutions.

In medical education and training, too, it is essential that teachers and students, but also patients, recognise the complex interrelationships of divergences in medical practice and the resulting stigma that must be removed. The DDS can serve as a basis for taking these interrelationships into account, for incorporating creative approaches to solutions and a diversity-sensitive attitude into the doctor-patient relationship and medical treatment. For example, the first and so far only World Report on Disability from 2011 noted a still existing negative infiltration of doctor-patient-interactions through stigmatization of persons with disabilities and deviations. Misunderstandings, lack of knowledge and wrong presettings can endanger the treatment [32].

In order to create the framework conditions for an appropriate consideration of diversity and disability in the program, it is necessary to impart six core competencies to prospective physicians [20]: If possible, this should always be designed in the respective training courses of all health care professions in a patient-centred manner, across all occupational groups and under the premise “nothing for us without us“ [1]. This corresponds to the principles of Disability Studies.

## Zusammenfassung

Die „Disability & Diversity Studies“ (DDS) sind Forschungsrichtungen, die sich, ähnlich der Sozialen Arbeit, mit gesellschaftlichen Ein- und Ausschließungsprozessen befassen. Dimensionen von Behinderung und Vielfalt können im individuellen Leben und gesellschaftlichen Zusammenleben von Menschen zu Benachteiligungen und Ungleichheiten führen. Die DDS untersuchen diese Ungleichheiten und identifizieren intersektionale Zusammenhänge zwischen Diversitätskategorien. Das Konzept der Intersektionalität eröffnet die Sicht auf die Ver-schränkung von Diversitäten, was die Verstärkung von Ungleichheiten und Mehrfachdiskriminierungen zur Folge haben kann: bspw. als Frau und als ethnische Minderheit. Der Ausgangspunkt ist demnach nicht die Differenzkategorie an sich, sondern der Kreuzungspunkt mehrerer Kategorien [5]. Dieses Wissen über kategorisierende(n) Einordnung und Ausschluss in ihrer Intersektionalität ist grundlegend für das Auflösen von sozialer, gesellschaftlicher, politischer und wirtschaftlicher Ungleichheit. Der Bachelorstudiengang DDS der Fachhochschule Kärnten, in dem die Disability und die Diversity Studies miteinander verknüpft wurden, zentriert sich um diese Forschungsrichtungen und erarbeitet daraus praktische Lösungsansätze.

Auch in der medizinischen Aus- und Weiterbildung ist es grundlegend, dass Lehrende und Lernende, aber auch Patient*innen erkennen, welche komplexen Zusammenhänge von Diversitäten in der medizinischen Praxis wirksam sind und welche daraus resultierenden Stigmata es abzutragen gilt. Die DDS können als Basis dienen, um diese Zusammenhänge zu berücksichtigen, kreative Lösungsansätze und eine diversitätssensible Haltung in das Ärzt*innen-Patient*innen-Verhältnis und die medizinische Behandlung einfließen zu lassen. So konstatierte der erste und bislang auch einzige Weltbericht über Behinderung aus dem Jahr 2011 eine noch bestehende negative Infiltration der Ärzt*innen und Patent*innen-Interaktionen durch Stigmatisierung von Personen mit Behinderungen und Abweichungen. Missverständnisse, fehlende Kenntnisse und falsche Voreinstellungen können die Behandlung gefährden [32].

Um die Rahmenbedingungen für eine angemessene Berücksichtigung von Diversität und Disability in den Studiengängen zu schaffen, ist es erforderlich, angehenden Ärzt*innen in ihren Ausbildungen sechs Kernkompetenzen zu vermitteln [20]: Wenn möglich sollte dies in den jeweiligen Ausbildungen aller Gesundheitsberufe berufsgruppenübergreifend stets patient*innenzentriert unter der Prämisse „nothing for us without us“ konzipiert sein [1]. Dies entspricht den Prinzipien der Disability Studies.

## 1. Introduction and problem definition

### 1.1. The need for knowledge about disability and diversity 

The experience of disrespect, lack of sensitivity and devaluation can lead people with disabilities to accumulate negative experiences with the health care system, to no longer seek professional medical care and finally (have to) rely on self-diagnosis and treatment. Doctors, on the other hand, often lack the expertise and skills to distinguish whether a need for treatment arises from the disability or from other diseases. A limited knowledge of the life situation of people with disabilities damages the quality of the doctor-patient relationship and the medical treatment. Often there are also communication problems with the affected persons, which prevent a comprehensive diagnosis and lead to delays in therapy. This is accompanied by insecure care on both sides, which ultimately leads to inequalities and exclusions in medical treatment [32]. It is therefore urgently necessary to incorporate knowledge from the Disability and Diversity Studies into medical education and training [7], [12].

Disability and Diversity Studies are research fields that deal with social inclusion and exclusion processes. The central question is which categories and dimensions of diversity currently lead to inequalities and disadvantages, which experiences persons self-experienced and which needs to make visible. On the one hand, this article focuses on explaining how the DDS work as scientific disciplines and what their basic professional orientations are. On the other hand, the arc is to be drawn to a diversity-oriented education and further training, which is transferable to different disciplines and professions, but focuses on medicine. It is therefore clear from the basic orientation of the DDS what contribution these avant-garde studies can make to diversity-sensitive medical education and further training and what elements should be included in medical studies in order to promote discussion of the topics of disability and diversity and to do justice to the patients concerned.

#### 1.2. Development and basic orientation of Disability and Diversity Studies

Disability Studies as an interdisciplinary research field has its origins in the US and British disability movements of the 1980s. The aim of the disability movements was to move away from the medical model to the social model of disability. In contrast to the first, which used applied sciences to reduce the individual situation of people to their physical “illness” or “disability”, the social model focuses on social disadvantages and barriers. The disability movements wanted to show that the “defects” are less individually determined, but that people are at the same time “handicapped” by social constellations and defined as “disabled”. The criticism is directed at a purely medically justified deviation from a predefined norm and the resulting therapisation of the affected persons. From this critical point of view, disability is a social construction and as such should be included in scientific discussions. Furthermore, cultural contexts play a role, i. e. it is examined how disability is currently defined and has been interpreted in the course of history [21], [31].

Analogous to the basic ideas of Disability Studies, the DDS program generates ideas for replacing forms of care that found their place in separate institutions with self-selected models. The affected persons themselves define their need for support and actively request assistance. The former stigmatising logics of “diagnosis” and “treatment” are being removed and modified in inclusive community-based settings (deinstitutionalisation and community care approaches). The DDS thus follow the UN Convention on the Rights of Persons with Disabilities and other central human rights documents. In place of intervention orientation, which in many cases is still the determining factor in social work, there is a tendency for the self-involved to be controlled. According to Udo Sierck, there is still a lot to be done in this regard, since not only the forms of support and institutions need to be redesigned, but also the social thought patterns that have developed over the centuries, which – even today – deny the affected persons their subject status and force them into the role of victim [28]. By conceiving of diversity categories as socially produced, the allocation of opportunities and obstacles associated with these attributions can be rearranged and previously closed “inclusion windows” can be opened for as many citizens as possible.

The Diversity Studies go back to the US-American civil rights movement, in which minority groups fought for their rights. The aim was to recognise diversity in society and to counteract discrimination. Especially for diversity management the anti-discrimination legislation should be seen as a source and driver. Initially, the categories of ethnicity, gender and age were at the centre of civil rights movements and Diversity Studies. In recent decades there has been a conceptual differentiation of diversity dimensions (age, gender, sexual orientation, physical impairments/disabilities, social and cultural background, rural and urban areas, social status etc.). A wide variety of research disciplines are concerned with diversity, so that these Studies have become an integrated research program, in which many directions, such as the economic and social sciences, cultural studies and the human medicine, “cooperate”.

Diversity categories are not problematic per se, because they allow us to orient ourselves in the world and classify phenomena. That means that our knowledge about the world is acquired and internalized [...] and is available to us as stable everyday knowledge and action orientation [4]. Social knowledge is naturalized through language, everyday communication and institutionalized language regulations and appears to us as objectively given [4]. Categories of order function mostly binary and as social “ushers”, so that we structure inclusion and exclusion, belonging and non-belonging through them [4]. 

Diversity categories transport fixed ideas, for example about “the disabled”, “the young”, “the homosexuals”. This can promote the maintenance of an unquestioned norm that distinguishes itself from the “others” as well as the perpetuation of stereotypes and prejudices about groups [18]. According to Roswitha Hofmann, the current diversity categories mostly name minorities, while the majority that makes this difference remains unmarked. For example, the category “age” often only addresses “older” persons, but not “younger” ones, and the category “sexual orientation” is usually only associated with homosexuals and not with heterosexuals, although both of them have an “age” or have a “sexual orientation”. […] Categorizations also promote thinking in oppositions (“we” and the “others”) [18]. By suggesting the possibility of clear allocation and the homogeneity of groups, differences of and between people are blurred. Concentrating on one category while ignoring or neglecting other categories can lead to exclusions and “blindness” [8]. According to Verena Eickhoff and Lars Schmidt, too, an intersectional analysis of differences and inequality relations is therefore to be favoured and categories are to be thought of as interdependent [8]^1^.

The term “diversity” marks diversity on the one hand, but on the other hand it comes into contrast with the indeterminability of humans when fixed ideas are addressed with it. Roswitha Hofmann remarks that the term “diversity” is never conclusively determinable itself [18]. Jacques Derrida had also drawn attention to this by replacing the “e” with an “a”; in the term “difference”. The replacement remains inaudible [6]. The term “différance” aims to keep the work open to diversity and to break the logic of negating the heterogeneous [6], [9]. Any use of the term “diversity” must – if one follows Derrida today – be conscious of categorizing something about the other that cannot be named. Shortening uses of the term “diversity” are confronted with human rights and context-sensitive issues in the studies of DDS.

## 2. Project description: The DDS program and inspirations for medicine

In the following, the DDS program including its curricular structure will be presented in order to gain inspiration from its content and to derive competencies that could play a role in medical education and training. A multidisciplinary approach is becoming increasingly important in the health and social science degree programs at Carinthia University of Applied Sciences in order to be able to react to changing working cultures and living environments. The joint study of different courses of study also becomes more relevant, since the later work often takes place in heterogeneous teams^2^. This points to the urgent need to implement cross-curricular and cross-professional forms of teaching and learning, since a health problem usually has a social dimension, such as social isolation.

### 2.1. The DDS program 

Social exclusion and marginalisation are changing due to changing working cultures, increased migration movements, activating social policy, neo-liberal economic practices, demographic change, etc, so that the required scientific and professionalism of DDS must always react to this change. The spectrum of tasks and fields of action of the DDS is becoming broader, above all due to the need to implement the UN Disability Rights Convention in all areas of society.

The DDS is a program that is based on the pulse of the present world, which – according to Gerhard Gamm – is no longer held together by any will [13]. In this “surreal world” there is no longer the one and correct reality, but its conflicting interpretations [13].

The DDS are in the middle of this ambivalence. On the one hand, they are seismographs of the present world and – to put it socio-philosophically – they capture their surrealities in thought [16]^3^. On the other hand, as a program at a university of applied sciences, they point beyond this analytical level and propose – with the participation of those concerned – suitable “solutions” for practical application. The focus is on assistance models that enable those affected to lead a self-determined and supported life according to their needs. Another field of action touched upon by the DDS is medical education and training and medical practice in general. When people interact, hierarchies and power structures are created, which cause inequalities and exclusions. In all contexts – also between doctors and patients – the successful handling of disability and diversity and the quality of the encounter with the other person always plays an excellent role.

**Figure 1:**
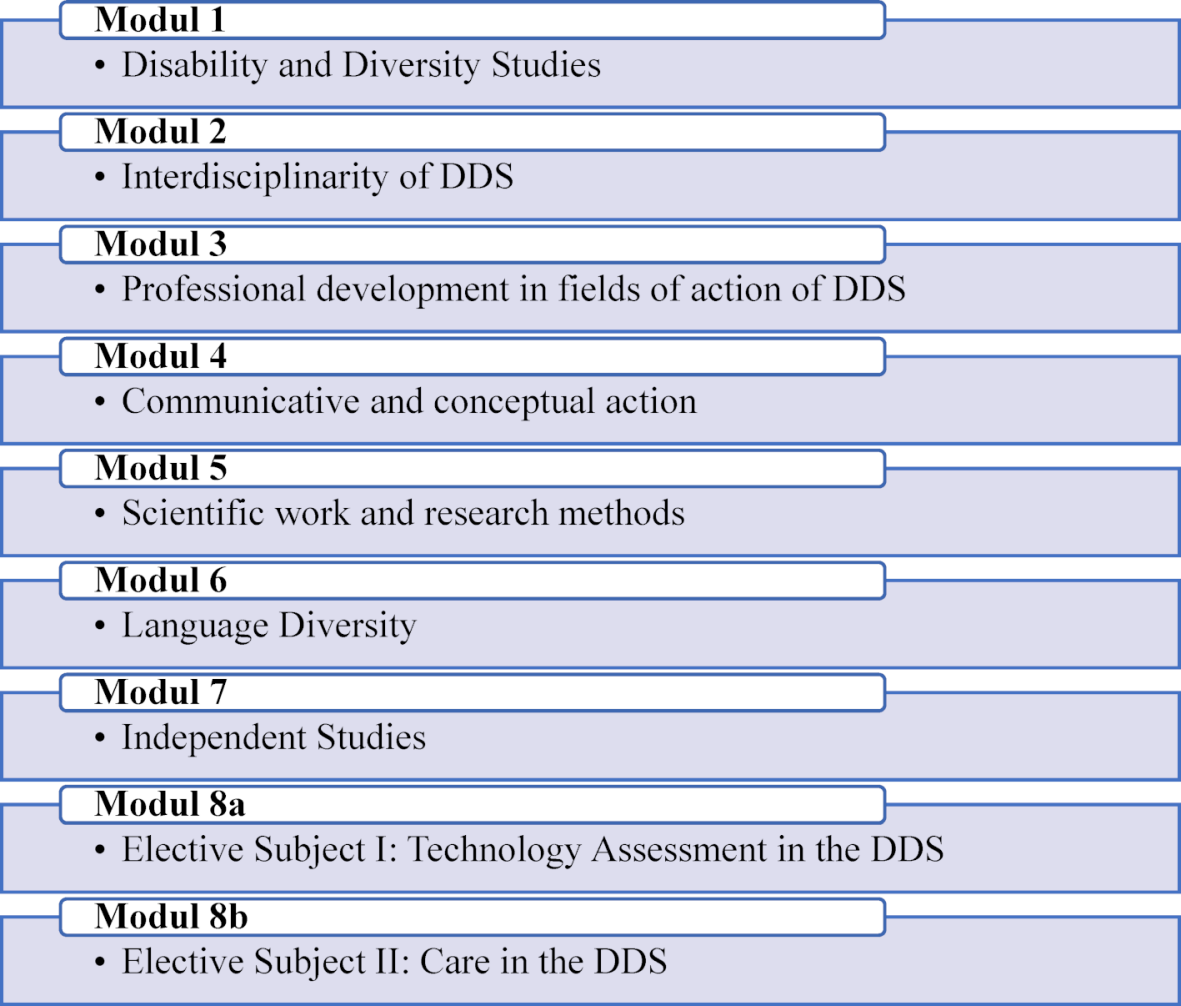
Module structure of the Bachelor's program Disability & Diversity Studies

The DDS program was established in 2012 and 2013 as a Bachelor's program at the Carinthia University of Applied Sciences in the field of Health and Social Sciences, because it was recognised that the topics of disability and diversity are forward-looking (see figure 1 (Fig. 1)). As a part-time course of study, DDS enables students to combine work, leisure, family and other areas of life. During the semester (15 weeks) there are attendance times twice a month (fridays, saturdays and mondays) and one full week per semester (monday to saturday). The program comprises six semesters of 30 ECTS each. The job- and family-friendly attendance times are supplemented by elements of online teaching and self-study, in which self-, project and group work is carried out. The results of these elements flow into the attendance times by means of discursive processing.

The program is particularly attractive for self-affected students. Students with disabilities, a migration background, from educationally disadvantaged backgrounds and other experiences of social disadvantage or biographical crises (such as Burnout). Students can place their own experiences of exclusion at the centre of their academic debate and professional development. The DDS address the heterogeneous needs and interests of the study participants without changing university requirements. In the sense of an inclusive university, the framework conditions for studying are designed in such a way that the requirements can be met by as many people as possible: for example, a buddy system has been set up (students support each other in learning how to work scientifically) or it is possible to complete the course of study on a part-time basis.

The DDS program always adapts its scientific and professionalism to the changing processes of exclusion and marginalization that are, for example, being experienced in the world of work, due to changes in working cultures or migration movements. Firstly, the adaptation takes place through the further development of the curriculum, but this change is also registered and thematically integrated in the individual courses. This inclusion is particularly in Module 1 “Disability and Diversity Studies” and Module 2 “Interdisciplinarity of DDS”. In these modules, students familiarize themselves with the scientific foundations of Disability Studies and Diversity Studies and deal with reference disciplines such as psychology, pedagogy, philosophy, sociology, medicine, law and economics. The teaching of theoretical principles is interlocked with practical examples to ensure a theory-practice transfer.

The spectrum of tasks and fields of action of DDS has become much broader in recent years. In Module 3 “Professional Development in the Fields of Action of DDS” this extends from the areas of education and work, art and culture, business and administration, through to management and entrepreneurship and above all the initiatives of the self-representation of those concerned. At the same time, developments in society as a whole are intermingled due to internationally binding ethical guidelines and interdependencies between social subsystems (social affairs, economy, medicine, justice, etc.) to complex fields of activity that increasingly require a multidisciplinary approach. This practical relevance is deepened in the two practical phases and accompanied by reflection events. Module 3 supports the students in reflecting on their professional actions to the extent that they themselves contribute to reproducing categories that include and exclude others. The “Competence Workshop-Course” is hereby starting in the first semester.

Social professions are not free to perpetuate marginalising attributions to others, for example by making them more vulnerable to the influence of others. Special offers for specific target groups (delinquents, drug addicts, mentally ill people, etc.) and separate them into different help lines depending on the problem [2], [4]. These ascriptions and the intersectionality of diversity categories have not been sufficiently taken into account so far – even by social professions. The DDS, as a social science program, explores the effectiveness of categories and underexposed power mechanisms that restrict people in their open vitality and diversity. According to Derrida, the term deconstruction stands for the attempt to question the unambiguous classification [6], [9]. This confrontation with one's own respectful or disrespectful attitude towards another person takes place in Module 3, but also in Modules 4 and 5, in which scientific work and participatory, stakeholder-controlled research methodology form the core.

In Module 4 “Communicative and Conceptional Action” students become familiar with the multi-professional and multi-disciplinary orientation of DDS and gain an overview of models of conversation, communication and intervention. This module enables students to sensitively adapt models of assistance and support to the needs of individuals and groups and to apply them in a situation-specific manner, taking into account the respective social space.

Module 6 “Language Diversity” offers students a choice of three languages: sign language, Italian, Slovenian and reflects the geographical location of the course in the Alpe-Adria region. Module 7 “Independent Studies” enables the so-called independent study. Various events from the tertiary education sector can be attended depending on the students’ interests. In addition, this module offers a lecture series with high-ranking academics and representatives from practice every semester.

The DDS program participates in productive social change around the concepts of “disability” and “diversity”. Promising new approaches, such as supported employment, assisted living, community care, personal assistance, peer counseling, ex-in movement in the field of psychiatry, which must be helped to be implemented. To this end, reference is also made to international discourses and developments. Module 8a and 8b in particular, in which two electives are offered (Technology Assessment in the DDS, Care in the DDS), take this topicality into account by focusing on both new support settings (Care) and the comprehensive mechanization of society (digitalization). Here DDS cooperates with the medical technology program at Carinthia University of Applied Sciences (especially with the Active Assisted Living work area).

#### 2.2. Possibilities and suggestions for curricular integration in the study of human medicine 

In order to minimise the gaps in doctors' knowledge mentioned at the beginning, a universal demand for the teaching of a set of skills is to be made by answering the following three questions [19]:

- Why is it important to train doctors about the care of people with disabilities?
- Why is there still a lack of disability-related training in medical education?
- How can future medical training be improved?

To answer these questions or in order to create the necessary conditions in the study courses for this, it is necessary to impart six core competencies to the future physicians during their training [20]:

1. Dealing with disabilities always in the context of human diversity throughout life and within the social and cultural environment.
2. Qualification trainings for the evaluation of disabilities and functional consequences of health conditions, taking into account the effects on treatment and care processes
3. Training in general principles of etiquette when dealing with people with disabilities.
4. Familiarising with the role of other health professionals to ensure the development of integrated teams to care for people with disabilities.
5. Understanding of the legal framework for the admission of disabled persons to health care facilities and the universal principles.
6. Acquisition of competence in patient-centered care approaches, including understanding the perception of the quality of life of patients.

Of these core competencies, patient centeredness (patient centeredness or people-centered services) as well as inter-professional care are particularly noteworthy. Thus the Alliance for Disability in Health Care Education also demands “Nothing about us without us!”. This basic principle should apply to the training of all health care professions, including the design of medical curricula. The inclusion of people with disabilities in the development of teaching content as well as in the implementation of curricula for future physicians can help to ensure that students acquire the competence to provide patient-centred care for people with disabilities. To this end, the alliance has currently developed six fields of competence to be anchored in the curricula:

1. Contextual and conceptual framework for disabilities
2. Professionalism and patient-centered care
3. Legal obligations and responsibilities for the care of patients with disabilities
4. Teams and system-based practice
5. Clinical evaluation
6. Clinical care over the life span and during changes.

The fields of competence are each preceded by a justification and a learning outcome and the field itself is operationalised concretely and clearly in five to ten sub-items [1].

## 3. Disability and diversity in the context of human medicine programs [results and perspectives]

The fields of competence make it clear how relevant the topics of disability and diversity are for all professions that work directly with people. This is especially true for medicine, as it deeply touches the physical and psychological integrity of a person.

At the medical faculties, only individual parts of this complex catalogue of requirements have been implemented or are in the curricula accordingly. For about five years, however, a growing number of publications have been appearing that present individual aspects of disability and diversity from the perspective of medical teaching. Sarmiento et al. describe a longitudinal anchoring of the field of diability in the first two years of medical studies. The overall aim was to develop a curriculum that teaches medical students about disability in a way that is based on the concerns, perspectives and experiences of people with disabilities [27]. One publication addresses both topics by describing the teaching of aspects of disability in a culturally sensitive competence context. The acquisition of competence should take place in a “culture of disability” in the areas of communication, patient and family ideas about health care. Folkloristic or non-traditional forms of treatment are also considered [26].

In summary, it should be noted that in the curricula of medical courses of study in the German-speaking countries, considerations on anchoring disability and diversity, although by no means general or even uniform, are now being implemented or are at least being implemented in a whole series of approaches [17], [24]. Until a few years ago, this was done only marginally in the form of courses in conversation management, medical sociology and of social medicine and public health. In addition to the consideration of individual aspects of disability and diversity in individual learning subjects, there are also far-reaching developments in medical curricula, in which aspects relating to gender and cultural diversity or disability have been incorporated separately [24], [25]. With regard to the above-mentioned demand for comprehensive competence profiles that include both diversity and disability in equal measure, and which are always conceived in a patient-centred way in the respective training courses of all health care professions, across all occupational groups and always under the premise “nothing about us without us”, far-reaching development work is still required. This should also be flanked by corresponding research, including ensuring that it is supported financially and with human resources.

## Notes

^1^ The DDS programme follows the more recent debates in Diversity Studies and thus points beyond the model of Lee Gardenswartz and Anita Rowe (1994).

^2^ A project is planned in which, for example Students of physiotherapy, health and nursing, DDS and social work study together and go through practical phases.

^3^ Georg Wilhelm Friedrich Hegel writes in his Grundlinien der Philosophie des Rechts (from the years 1832-1845): That which is to be understood is the task of philosophy, for that which is is reason. […] Here is the rose, here dances (Hegel 1986, p. 26).

## Competing interests

The authors declare that they have no competing interests.
